# Transcriptomic signature of drought response in pearl millet (*Pennisetum glaucum* (L.) and development of web-genomic resources

**DOI:** 10.1038/s41598-018-21560-1

**Published:** 2018-02-21

**Authors:** Sarika Jaiswal, Tushar J. Antala, M. K. Mandavia, Meenu Chopra, Rahul Singh Jasrotia, Rukam S. Tomar, Jashminkumar Kheni, U. B. Angadi, M. A. Iquebal, B. A. Golakia, Anil Rai, Dinesh Kumar

**Affiliations:** 1 Centre for Agricultural Bioinformatics (CABin), ICAR-Indian Agricultural Statistics Research Institute, New Delhi, India; 2 Department of Biochemistry and Biotechnology, Junagadh Agricultural University, Junagadh, Gujarat India

## Abstract

Pearl millet, (*Pennisetum glaucum* L.), an efficient (C4) crop of arid/semi-arid regions is known for hardiness. Crop is valuable for bio-fortification combating malnutrition and diabetes, higher caloric value and wider climatic resilience. Limited studies are done in pot-based experiments for drought response at gene-expression level, but field-based experiment mimicking drought by withdrawal of irrigation is still warranted. We report *de novo* assembly-based transcriptomic signature of drought response induced by irrigation withdrawal in pearl millet. We found 19983 differentially expressed genes, 7595 transcription factors, gene regulatory network having 45 hub genes controlling drought response. We report 34652 putative markers (4192 simple sequence repeats, 12111 SNPs and 6249 InDels). Study reveals role of purine and tryptophan metabolism in ABA accumulation mediating abiotic response in which MAPK acts as major intracellular signal sensing drought. Results were validated by qPCR of 13 randomly selected genes. We report the first web-based genomic resource (http://webtom.cabgrid.res.in/pmdtdb/) which can be used for candidate genes-based SNP discovery programs and trait-based association studies. Looking at climatic change, nutritional and pharmaceutical importance of this crop, present investigation has immense value in understanding drought response in field condition. This is important in germplasm management and improvement in endeavour of pearl millet productivity.

## Introduction

Pearl millet, (*Pennisetum glaucum* L.) cereal crop is well known for its hardiness and grown in arid and semi-arid tropical regions of Asia and Africa. It is a short duration annual, cross pollinated, C4 panicoid plant having diploid (n = 7) genome with draft assembly genome size ~1.79 Gb^1^. Millet is the most important crop for biofortification which is highly relevant in combating global micronutrient malnutrition. Millet being medium GI (Glycemic Index) cereal, is diabetic friendly^2^. Looking at pearl millet’s highest productivity (3.3 tonnes per hectare) and global average productivity (0.83 tonnes per hectare), it can be deduced that this promising crop has genome plasticity to increase its productivity at least by four fold^3^. Pearl millet has 11.8 g of proteins providing 363 Kcal of energy relatively higher that rice or wheat^4^. Attributes of pearl millet like rich in nutrients, high energy, less starch, high fibre (1.2 g/100 g, predominantly insoluble), and higher α-amylase activity (8–15 times) than wheat, make pearl millet globally relevant crop produce^5^.

Millet is also used as fodder. Being C4 plant, it is efficient biomass producer even with less water requirement making it further attractive crop especially in regime of climate change. Pearl millet is also used in making breads and cookies, for ethanol production in industries, used as poultry and animal feed and biofuel crop^6^. Since it is rich in antioxidants, it is used in nutraceutical industry^7^.

In a study projecting climate change till 2099, it is indicated that there will be extreme increase in drought selectively in Western Hemisphere (major part of Eurasia and Africa) but there will be contrarily high moist regions, from Alaska to Scandinavia^8^. Adversely, drought affected region also covers major pearl millet growing area decreasing the productivity at least by 25%^9^. In such geoclimatic situation, improved millet variety with further drought tolerance would be a preferred crop due to its high photosynthetic efficiency with low transpiration as C4 plant^10^.

Limited number of Quantitative Trait Loci (QTLs)^11^ and genomic resources are reported in pearl millet. The reported genomic resources of pearl millet are in the form of simple sequence repeats (SSRs)^12^, expressed sequence tags (EST)^9,13^, EST-SSR^14–17^, ISSR^18^ and SCAR^19^. Gene-based single nucleotide polymorphism (SNP) and Conserved-Intron Scanning Primer (CISP) marker^20^ and DArT markers^21^. None of these are available in the form of web-based genomic resources. Looking at genome size, these markers are too less and moreover till now there is no trait specific genic region marker discovery using transcriptomic approach. Approaches like marker-trait associations, genomic selection, genome sequence and genotyping-by-sequencing are still warranted^22^. Genomic resources in pearl millet and management of abiotic and biotic stress are required to have insights of functional genes enabling crop improvement by molecular breeding approaches^23^.

Earlier work were based on heat treatment in phytotran^13^ and chemically induced drought using hyperosmotic polyethylene glycol (PEG)^9^ for induction of drought. All these were based on subtractive hybridization and cDNA (complementary Deoxyribonucleic Acid) library approach thus yielding very limited number of differentially expressed transcripts (<500)^13^. Moreover, tissues selected in previous experiments were confined to leaves only. For water and nutrient intake, root systems are most critical^24^ thus they should also be included for better holistic view at physiological and molecular level. Moreover, root senses and responds to drought first^25^ and mediates stress signals for root biomass adjustments, anatomical alterations, and physiological acclimations^26^ thus transcriptomic investigation of root tissue is much more required^27^.

Since the effect of drought on root and leaf varies significantly in millets^28^, thus specific tissue approach is imperative for identification of pathway and marker discovery. None of these experiments were based on withdrawal of experiment which truly mimics the actual drought condition of field.

Transcriptomic approach of a single crop genotype with drought treatment has been reported successfully in tea plant deciphering major pathways regulating drought tolerance^29^. Such single genotype-based transcriptomic analysis has been successfully used to delineate major physiological response against drought in tomato^30^ and cassava^31^ also. In case of field crop like soybean, water deficit response has been found variety/ accession specific^32^. Since single genotypes-based experiment has been found more holistic in understanding the basic physiological mechanism operating at species level, thus such experiments are needed in investigation of drought response in pearl millet species also. Transcriptome database is available for large number of crops but no such database has been developed for orphan crop like pearl millet to be used as genomic resources in crop improvement research. Since whole genome of the pearl millet has recently been available^1^, thus development of such web resources can be done advantageously with SNP discovery by comparing with reference sequence along with genome annotation analysis. Such genic region putative markers (SSRs, SNPs and InDels) discovery can be rapid and cost effective which can be used in drought trait improvement program in future for better productivity of pearl millet.

The present study aims at identification of differentially expressed genes in leaf and root tissue of millet in response to drought induced by withdrawal of irrigation in the field. It also aims at identification of transcription factors (TFs), genic region putative markers *viz*., SSRs, SNPs and Indels (Insertions and Deletions), gene regulatory network (GRN) having hub genes and development of web-genomic resources.

## Material and Methods

### Seeds sowing in green house and drought treatment with recording of soil parameters

Drought tolerant pearl millet J-2454 variety was sown in greenhouse during summer season of the year 2014-2015 at Agriculture Farm of Junagarh Agriculture University, Gujarat, India (21.5222° N, 70.4579° E, 107 meter above mean sea altitude). Seeds were sown in 2 kg polythene plastic bag under small greenhouse and polythene bag fill with equal weight soil mixture of sand, vermicompost and FYM in ratio of (40:40:20) and 25 to 30 seeds sown per polythene bag with three replication of one genotype to comparative study with control and water stress (or water withhold). Soil parameters, namely, average pH, electrical conductivity (EC), Maximum water holding capacity were recorded. Parameters were also recorded for water used in irrigation. During experiment in greenhouse condition day and night temperature and relative humidity (RH) were recorded. Seedlings were maintained by thinning post 10 days after sowing (DAS). Drought conditions were created by withdrawal of irrigation for 6 days after 23rd day of sowing of millet. Leaf and root tissues of pearl millet were collected after 6 days of withdrawal of irrigation (i.e., 29^th^ days after sowing) from control and drought treated plants. Withdrawal of irrigation at different time point can be an artificially created drought best suited by gene expression profiling in field crop^33^. In control plant, regular irrigation interval of alternate day was maintained. Major physiological information can be seen at transcriptomic level on 29th day of sowing as reported in other millet crop species^34^. Sampling at 29th day of sowing were as per the leaf and root tissues (two sets each) were collected from control and drought induced plants for transcriptomic studies. Tissues were frozen in liquid nitrogen and stored at −80 °C.

### Plant tissue collection and RNA extraction

In order to minimize variability across samples, sample pooling approach was followed by taking ten biological replicates of both tissues, *viz*., root and leaf^35^. Total RNA was isolated from these tissues under control and drought condition using the standard protocol of *TRIZOL* RNA isolation^36^. It was further purified with Magnetic Oligo (dT) beads in accordance to the manufacturer’s instructions (Life Technologies, Grand Island, NY).

### Pre-processing, *de novo* assembly and identification of differentially expressed genes (DEGs)

The single-end Illumina reads were generated using root and leaf RNA of pearl millet genotype J-2454 having approximately 5678956 million and 6463411 million single-ends reads under normal and drought stress condition. The raw reads were pre-processed to remove any adaptor contamination using trimmomatic^37^ software with parameters read length ≤36, poor quality ≤3 and HEADCROP:10 bases. These pre-processed data were used for transcriptome assembly along with the identification of differentially expressed genes and other analysis, transcription factor identification and putative genic markers’ prediction. The different combinations used were root control vs. root under drought (RC, RT), leaf control vs. leaf under drought (LC, LT), root control vs. leaf control (RC, LC) and root under drought vs. leaf under drought (RT, LT).

The pre-processed high-quality reads were assembled using Trinity platform^38^. The abundance estimation for transcripts obtained was performed using ‘RNA-Seq by Expectation-Maximization (RSEM)^39^. For the above mentioned transcriptome datasets, differentially expressed genes were identified using edgeR package^40^ of Bioconductor. The significant DEGs were obtained with stringent parameters taking fold change value as two and FDR (False Discovery Rate) <0.05^41^.

### Functional Annotation of transcriptome assembly

The sequence similarity search was conducted against the National Center for Biotechnology Information (NCBI) non redundant protein (Nr) database, and Swiss-Prot protein database using the BLASTx algorithm specifying E-values of less than 10^−3^. BLAST2GO^42^ was used for Gene Ontology (GO) categorization and functional enrichment pathway analysis.

Transcription factors (TFs) and cofactors play very important roles in the expression of genes. Differentially expressed genes from all the combinations, i.e. (RC, RT), (LC, LT), (RC, LC) and (RT, LT) were considered for identification of TFs against PlantTFDB 3.0 with threshold e-value 1e-3^43^.

### MicroRNA (MiRNA) binding site prediction

PsRNATarget server^44^ was used for prediction of microRNA targets against all the mature microRNAs of Poaceae family *viz., Aegilops tauschii, Brachypodium distachyon, Elaeis guineensis, Festuca arundinacea, Hordeum vulgare, Oryza sativa, Sorghum bicolor, Saccharum officinarum, Saccharum sp., Triticum aestivum, Triticum turgidum, Zea mays*. We used all the 4 sets of comparison, viz., (RC, RT), (LC, LT), (RC, LC) and (RT, LT) to predict miRNA targets.

### Gene Regulatory Network Analysis

The highly differentially expressed genes with fold value ≥8 from up and down regulated gene were considered for the gene interaction network. Network were visualized and carried out for further analysis using Cytoscape (version 3.2.1)^45^ which is an open source platform for visualizing complex networks. ARACNE (Algorithm for the Reconstruction of Accurate Cellular Networks), a novel algorithm, specifically designed to scale up to the complexity of regulatory networks operating in the living cells was used. Network Analyzer plug-in was used to analyse the network centrality. The plug-in computes specific node centrality parameters and describing the network topology. Hub genes of complex networks were also obtained according to analysis of degree, betweenness and stress. The genes at the top of degree, betweenness and stress distribution were defined as hub genes.

### Identification of SNP and SSR in Pearl Millet transcriptome

For identification of putative genic SSRs from both assembled transcripts and differentially expressed transcripts, MISA^46^ (Microstellite Analysis) tool was used. Further, Primer3^47^ was used for designing primers with parameters like annealing temperature (Tm) min: 57 °C, optimal: 60 °C, and maximum: 63 °C, primer size min: 15, optimal: 18, and maximum: 28 oligo-nucleotides.

Variant discovery (SNP and InDels) was done by both approaches namely, comparison of transcripts with de novo transcriptome assembly of genotypes J-2454 and also with available reference sequence of millet (genotype 23D2B1-P1-P5). For reference sequence based SNP discovery, genomic data of pearl millet *Cenchrus americanus* was downloaded from NCBI (https://www.ncbi.nlm.nih.gov/assembly/GCA_002174835.1/). Circos tool was used for generation of circular map of variants^48^.

Reads were mapped using BWA-0.7.5a (Burrows-Wheeler Aligner) and Samtools^49^. The SNPs were identified at read depth (d) ≥8 and quality depth (Q) ≥20^50^. Using the SAMtools program “*vcfutils*”, SNP sites were further filtered, based on the criteria of 90 bp on both sides of the SNP in the alignment to ensure it in exon^51^.

### Validation and Expression Analysis by qRT-PCR

The first strand cDNA was synthesized from an aliquot of total RNA for each sample using RevertAid First Strand cDNA Synthesis Kit (Genetix, USA) and served as template for qRT-PCR (Quantitative Real Time Polymerase Chain Reaction). For quantitative PCR, 13 transcripts (6 for leaves and 7 for root) were randomly selected for primer designing using Primer 3 software^46^. The qRT-PCR was performed using QuantiFast SYBR Green PCR Master Mix (Genetix, USA) on ABI-7300 Real-Time PCR detection system, (Applied Biosystem) using standard 40 cycles along with melt curve step. Housekeeping gene, actin was used as endogenous reference for normalization. To obtain linear relationship, PCR conditions were optimized for each set of gene. Finally, differential gene expression were computed in terms of ΔΔCT fold change value^52^.

### Web-genomic resource development

Pearl millet transcriptome database (*PMDTDb*) catalogues the information related to assembled contigs or transcripts, DEGs, the pathways in which these are involved, detailed SSR markers, and variants such SNPs and indels and miRNAs. It has *three-tier architecture*, i.e., client tier, middle tier and database tier. Web pages are developed for browsing the database along with the queries by user in client tier. All the information regarding contigs, markers, variants etc. are arranged in tables in various tables in MySQL in the database tier. For execution and fetching of user’s query, scripting is done in PHP (Hypertext Preprocessor) in the middle tier. The pearl millet web-resources is available at http://webtom.cabgrid.res.in/pmdtdb/.

## Standard of Reporting

### Resource Identification

Germplasm resource used in the studies are completely disclosed by name of variety.

### Gene Nomenclature

HGNC is followed by default.

### Availability of Supporting Data

The transcriptome dataset of the study used in this article are available in the NCBI repository with following accessions and is kept at hold till the publication. These would be made public after publication.

Bioproject: PRJNA385901

Biosamples: SAMN06920424, SAMN06920426, SAMN06920432, SAMN06920433

SRA accession number: SRR5839373, SRR5839374, SRR5839375, SRR5839376

Since data is generated in the studies and kept in public domain, thus there is no violation of Fort Lauderdale and Toronto agreements.

## Results and Discussion

### Greenhouse conditions and soil parameters

Soil and weather parameters were recorded. Irrigation withdrawal method was used successfully to induce drought. Average pH and EC of soil mixture were found to be-7.37** ± **0.01 and-1.18 ± 0.01 (ms), respectively. Maximum water holding capacity of soil mixture was found to be 30.15 ± 0.28%. Average pH and EC of water used in irrigation were found to be −7.07 ± 0.14 and **−**0.36 ± 0.01 (ms), respectively. Maximum day temperature (36–37 °C) and minimum night temperature (24–25 °C) were recorded along with day (84–86%) and night (49–51%) humidity. Other soil parameters and weather information are given in the Table 1.

**Table 1:** Soil Parameters and Weather Information.

Replication	R1	R2	R3	Average ± SD
**Soil pH, EC and maximum water holding capacity (MWHC %)**				
Soil – pH	7.36	7.38	7.37	7.37 ± 0.01
Soil – EC	1.19 (ms)	1.18 (ms)	1.19 (ms)	1.18 ± 0.01 (ms)
MWHC (%)	29.90	30.10	30.45	30.15 ± 0.28
**Water pH and EC**				
Water – pH	7.20	6.92	7.10	7.07 ± 0.14
Water – EC	0.36 (ms)	0.37 (ms)	0.36 (ms)	0.36 ± 0.01 (ms)

### Pre-processing and *de novo* assembly of transcriptomic data

Transcriptome data was generated successfully from both set of tissue samples, *viz*., leaf and root. A total of 12142367 SE reads were generated representing 2272632 (root, control), 3360164 (root, treated), 3406324 (leaf, control) and 3103247 (leaf, treated) reads. After data cleaning and quality assessment at Q ≥25 for control samples and Q ≥35 for treated samples, a final dataset was obtained. A total of 7563927 SE reads were represented by 1980048 (root, control), 2154770 (root, treated), 1216328 (leaf, control) and 2212781 (leaf, treated) reads. *De novo* transcriptome assembly using different assemblers *viz*., Trinity, MIRA, Cap3 and CLC revealed trinity to be the best one (N50 = 949, assembly size ~46.26 MB) and was considered for further analysis (Table 2).

**Table 2:** Summary of *de novo* assembly of *Pennisetum glaucum* L.

	**Trinity**	**Mira**	**CAP3**	**CLC**
Total size of contigs (bp)	46259620	86542001	39881482	50,938,923
No. of contigs	95017	287045	133171	235,000
Longest contig	7397	5091	3328	5097
Shortest Contig	40	201	45	15
Mean contig size	487	301	299	217
N50 contig length	949	583	335	259

### Identification of Differentially Expressed Genes (DEGs)

**Figure 1:**
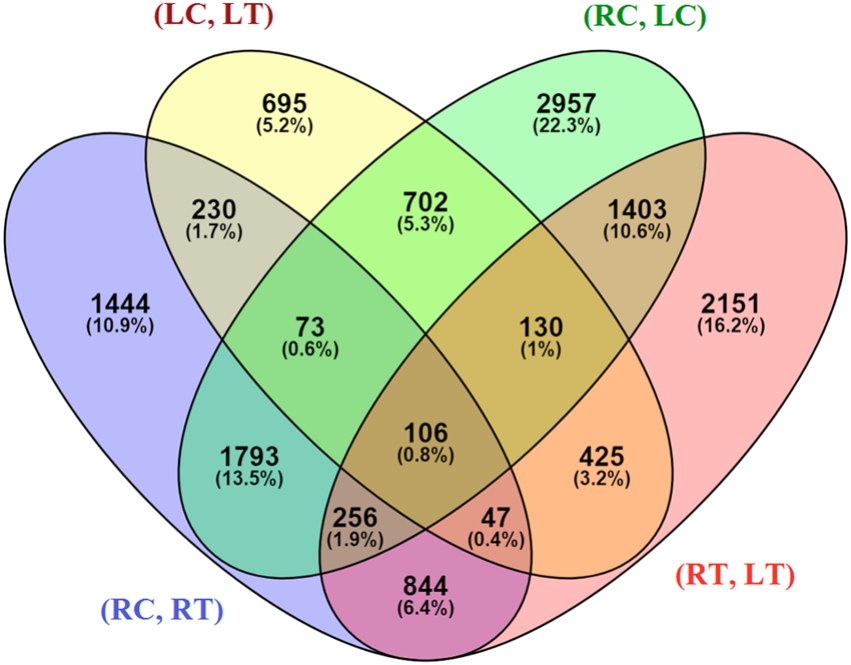
Venn diagram showing shared and unique DEGs of millet root and leaf transcriptome leaf transcriptome.

The analysis was conducted on four datasets, i.e., root control vs. root under drought (RC, RT), leaf control vs. leaf under drought (LC, LT), root control vs. leaf control (RC, LC) and root under drought vs. leaf under drought (RT, LT). The calculated read counts for a feature or gene was normalized to reads per kilobase million. A total of 4793, 2408, 7420 and 5362 DEGs were found in 4 sets of comparison, *viz*., (RC, RT), (LC, LT), (RC, LC) and (RT, LT), respectively in response to drought stress with FDR and corrected P-values of less than 0.05 (Additional file 1). Number of differentially expressed gene with their minimum and maximum logFC (Logarithm Fold Change) values are reported in Table 3. Shared and unique DEGs are depicted in the Venn diagram (Fig. 1) which shows that root is having more unique differential expressed genes (1444) with respect to leaf (695) in response to drought. A total of 106 genes were found common in all the 4 sets of comparison *viz*., (RC, RT), (LC, LT), (RC, LC) and (RT, LT).

**Table 3:** Number of up- and down-regulated differentially expressed genes in various sets (values in parenthesis are the their minimum and maximum logFC values).

Combinations	Differential expressed genes in 4 sets of root and leaf tissues comparison	
	Up-Regulated	Down-Regulated
(RC, RT)	1919 (2–13)	2874 (2–12)
(LC, LT)	1626 (2–12)	782 (2–11)
(RC, LC)	2977 (2–11)	4443 (2–13)
(RT, LT)	3043 (2–12)	2319 (2–13)

After quantification of expressions of each transcript for all the four sets of data, heatmap of differentially expressed transcripts were plotted along with MA plot and Volcano plot generated by edgeR (Supplementary Figure 1). Expression values in terms of Fragments Per Kilobase of transcript per Million mapped reads (FPKM) were log2 transformed followed by median-centered by transcript. The red dots in the plots represent true differentially expressed genes.

### Functional Annotation of transcriptome assembly

**Figure 2:**
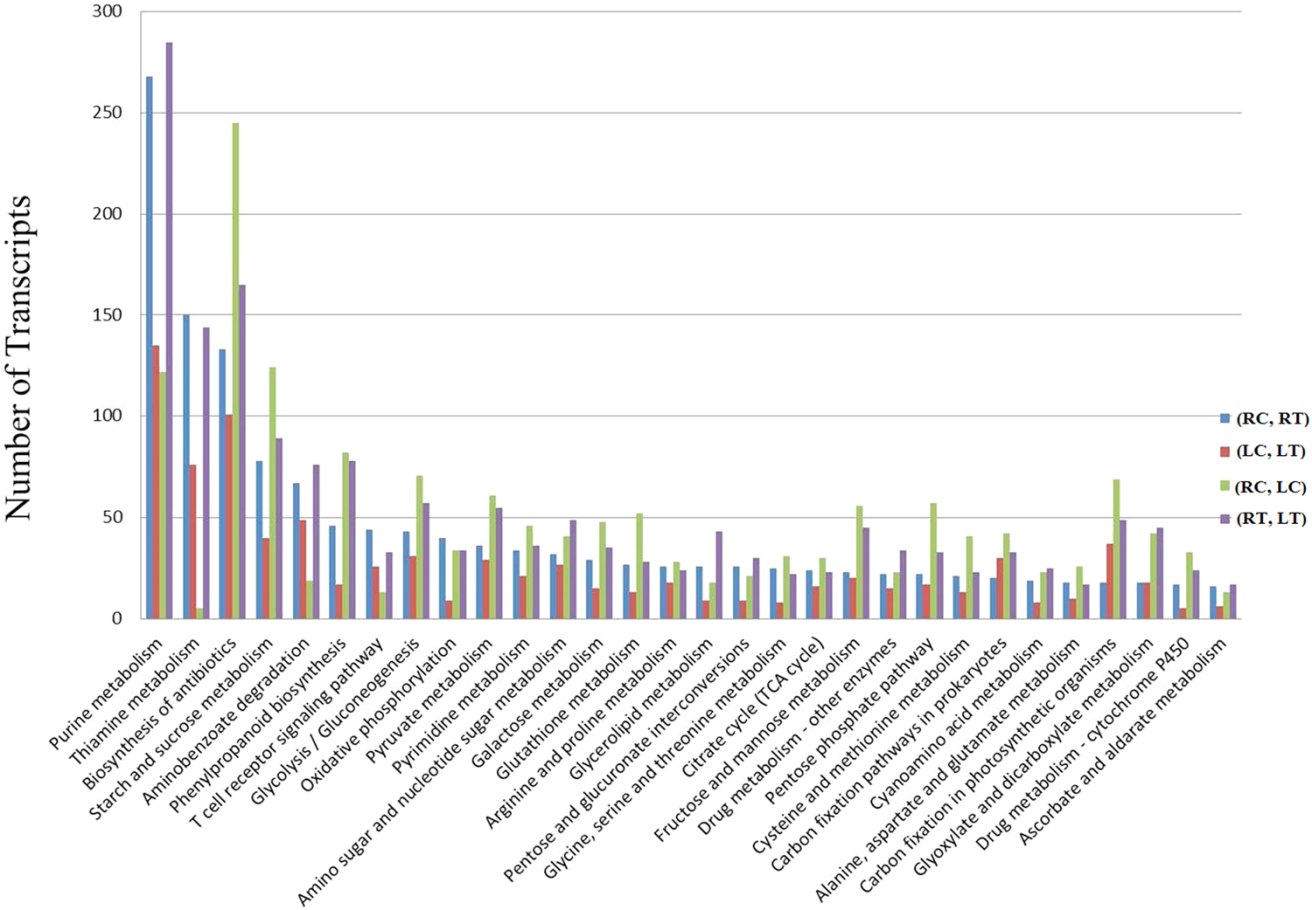
Pathway classification of the top 30 Common pathways in all the four sets of comparison.

As per Kyoto Encyclopedia of Genes and Genomes (KEGG) database, from a total of 4793, 2408, 7420 and 5362 DEGs obtained from (RC, RT), (LC, LT), (RC, LC) and (RT, LT) respectively, having their corresponding enzyme commission numbers were assigned to 121, 98, 126 and 117 KEGG pathways (Additional file 2). Out of these, 89 pathways were found common to all the four sets. The pathways most represented by contigs were purine and thiamine metabolism, biosynthesis of antibiotics, starch and sucrose metabolism, aminobenzoate degradation, glycine, serine and threonine metabolism and phenylpropanoid biosynthesis. Our analysis of top 30 common pathways reveals that leaf tissue are showing less metabolic activity with respect to root. This reflects that root has more DEGs than leaf for energy production and production of metabolites in response to drought for survival (Fig. 2).

Higher purine metabolism observed in KEGG analysis is due to role of purine metabolism in Abscisic acid (ABA) accumulation mediating abiotic stress pathway of plant defence^53^. KEGG pathway analysis further reveals that, tryptophan metabolism was conspicuously high in both leaf and root tissues in response to drought. Abiotic stress in crop leads to accumulation of proline along with other amino acids. This is because of tryptophan’s multifaceted role as osmolyte, ion transport regulation, stomatal control, detoxification reactions and redox-homeostasis^54^. This pathway analysis also reveals higher aminobenzoate degradation in both the tissues. Such higher degradation has been reported in abiotic stress of other crop like soybean^55^. This degradation increases the proline concentration in cell to protect cell from water deficit or abiotic stress. In case of drought resilience by rehydration there is both down regulation of proline biosynthetic pathway enzymes as well as upregulation of proline degrading enzymes^56^.

### Functional classification: GO annotation

GO classification of millet gene lists from (RC, RT), (LC, LT), (RC, LC) and (RT, LT) were also carried out. A total of 45 (20 BP, 13 MF and 12 CC) GO terms were assigned for (RC, RT), 40 (18 BP, 11 MF and 11 CC) for (LC, LT), and 43 (20 BP, 11 MF and 12 CC) for (RC, LC) and 50 (20 BP, 12 MF and 18 CC) for (RT, LT) (Supplementary Figure 2A–D). This GO classification reveals that under response of drought, root tissue has higher expression of signalling ((RC, RT): 239; (LC, LT): 115) which is due to role of root in sensing the drought first (rather than leaf) and mediating various signalling pathway of abiotic stress^25^. More growth activities in root with respect to leaf ((RC, RT): 60; (LC, LT): 28) is an adaptive response of plant where more root growth is expected for more absorption of water and slow growth of leaf is expected for water retention and energy balance in response to drought^57^. Higher abundance of immunity related transcripts ((RC, RT):52; (LC, LT): 34) in root is due to more defensive role of root in drought stress especially for pathways involved in detoxification and scavenging of free radicals^58^.

Similarly, regarding molecular function, under drought response, root tissues delineated higher catalytic activity ((RC, RT):1617; (LC, LT):952). Drought increases catabolic activity to produce and accumulate osmolytes like sugars, polyols, betaines and proline^59^. Similarly, higher transporter activity ((RC, RT):228; (LC, LT):119) in root is due to increase in nitrate, sodium and potassium transport activity under water deficiency stress^60,61^. Drought also increases the antioxidant activity in root ((RC, RT):29; (LC, LT):15) for protection of tissues from free radicals^62^.

Under the cellular component, the response of root and leaf against drought showed that cell ((RC, RT): 2035; (LC, LT): 1272), organelle ((RC, RT): 1707; (LC, LT): 1080) and membrane ((RC, RT): 816; (LC, LT): 524) is higher for root. Again, this is due to higher cellular activity of root in response to drought. Such cellular activity involves cell organelles like mitochondria and glycosomes for catabolic metabolism. Higher membrane activity is inevitable for such cellular and catabolic activity^25^.

RAB gene reported to mediate polar root growth in response to drought stress in common bean^57^ was found with highest magnitude among the upregulated genes in root. Similarly the role of other observed DEGs like serine acetyltransferase in sulfur assimilation pathway^63^, DHN9 (dehydrin 9) in membrane stabilizion, heat-shock gene (HSP17.8) and dehydrin 3 (DHN3) are involved in drought tolerance by controlling stomatal closure through controlling carbon metabolism^64^. We also observed differential expression of genes involved in energy balance and anti-oxidant activities which are already reported in drought response by various crops like SRP (stress responsive proteins) and DHN5 gene, controlling osmotic stress in wheat^65^, peroxidase controlling Reactive Oxygen Species (ROS) and antioxidant activities detoxifying. In fact SRP gene controls various other stress responsive genes^66^.

ROS concentration is increased in response to drought which may lead to cellular damage but gets detoxified due to antioxidant activities of peroxidase gene^58^.

In drought response, there is change in energy balance and metabolic pattern. Genes controlling these activities were found differentially expressed in our dataset, for example, LEA (Late Embryogenesis Abundant) gene reported to be associated with salt stress^67^, ATP (Adenosine Triphosphate) citrate synthase reported to control energy balance in drought^68^, aspartate kinase-homoserine dehydrogenase reported to control tricarboxylic acid (TCA) cycle^69^, glycolysis and Krebs cycle controlled by NADP dependent malic enzyme gene^70^. High affinity of nitrate transport uptake has been reported in drought treated roots and the same was observed in differential expression of nitrate transporter gene^59^.

### Transcription Factors identification

We obtained a total of 1757, 1056, 2826 and 1957 transcription factors in 4793, 2408, 7420 and 5362 DEGs from the ((RC, RT), (LC, LT), (RC, LC) and (RT, LT) sets, respectively (Additional File 3). We found 52 TFs common among 4 sets of root and leaf tissues comparison after removal of duplicates. These TFs can be used in drought and heat tolerance. Some of our TFs are already reported in other crops along with its association with abiotic stress, for example, WRKY in wheat^71^ and NAC1 in barley^72^.

A total of 7595 differentially expressed transcription factors have been found having binding site in 5022 transcripts (after removal of duplicate transcripts). We observed more number of TF with respect to number of DEGs. Potential reason for this could be due to one gene having multiple TFs^73^, overlapping of sequences^74^ and computational stringency^75^. TFs and miRNAs are two regulatory circuits coordinating transcriptional and post-transcriptional control of targeted genes^76^.

### MiRNA binding site prediction

**Figure 3:**
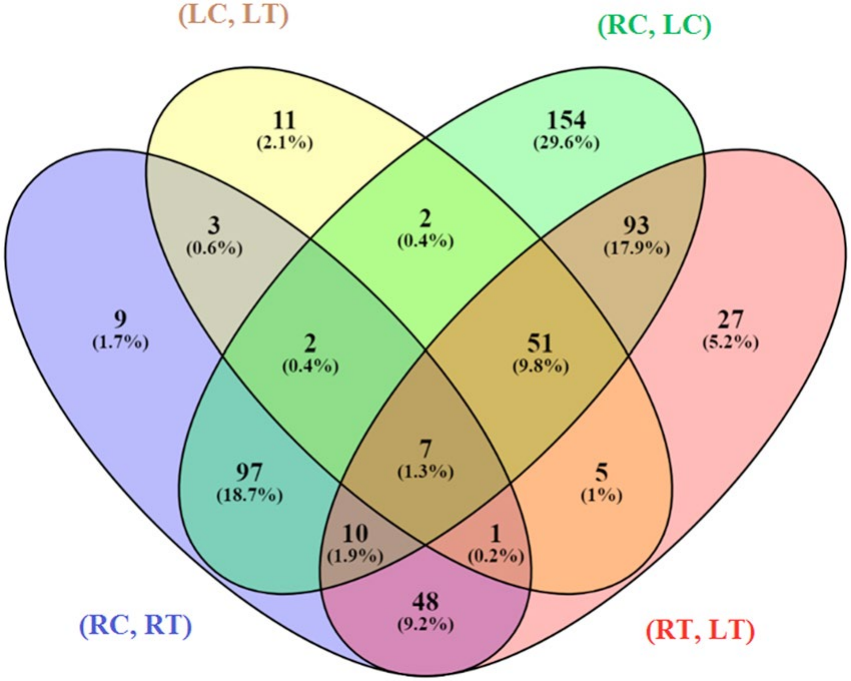
Venn diagram of common miRNAs.

A total of 267, 90, 827 and 445 mature miRNAs which targeted differentially expressed genes from 4 sets of comparison, *viz*., (RC, RT), (LC, LT), (RC, LC) and (RT, LT), respectively were predicted (Additional file 4). After removal of duplicates, we get 177, 82, 416 and 242 miRNAs and 61, 37, 105 and 73 target sequences for (RC, RT), (LC, LT), (RC, LC) and (RT, LT), respectively. We found 7 miRNAs *viz*., ssp-miR444a; osa-miR169d; ssp-miR444b.2; bdi-miR169l; osa-miR444f; osa-miR414; hvu-miR169 common in all the four sets (Fig. 3). This is due to wide conservation of miRNA having diverse functions in seedling growth and also in response to abiotic stress^77^. Few miRNAs may have multiple targets of different genes in the regulatory networks^78^. Thus, this is obviously expected with at least in some extensively conserved miRNA we may get but still they may have diverse functions.

miR444a is reported to mediate drought stress for its adaptation in wheat. It is also known to interact with MADS-box transcription factors, which is associated with stress response^79^. Besides, miR444 responded to drought stress in Dongxiang wild rice^80^. miR169d plays role in fighting drought stress in cotton^81^ as well as sorghum^82^. Also, miR169 family responds differently to drought in plants^83^.

### Gene Regulatory Network Analysis

**Figure 4:**
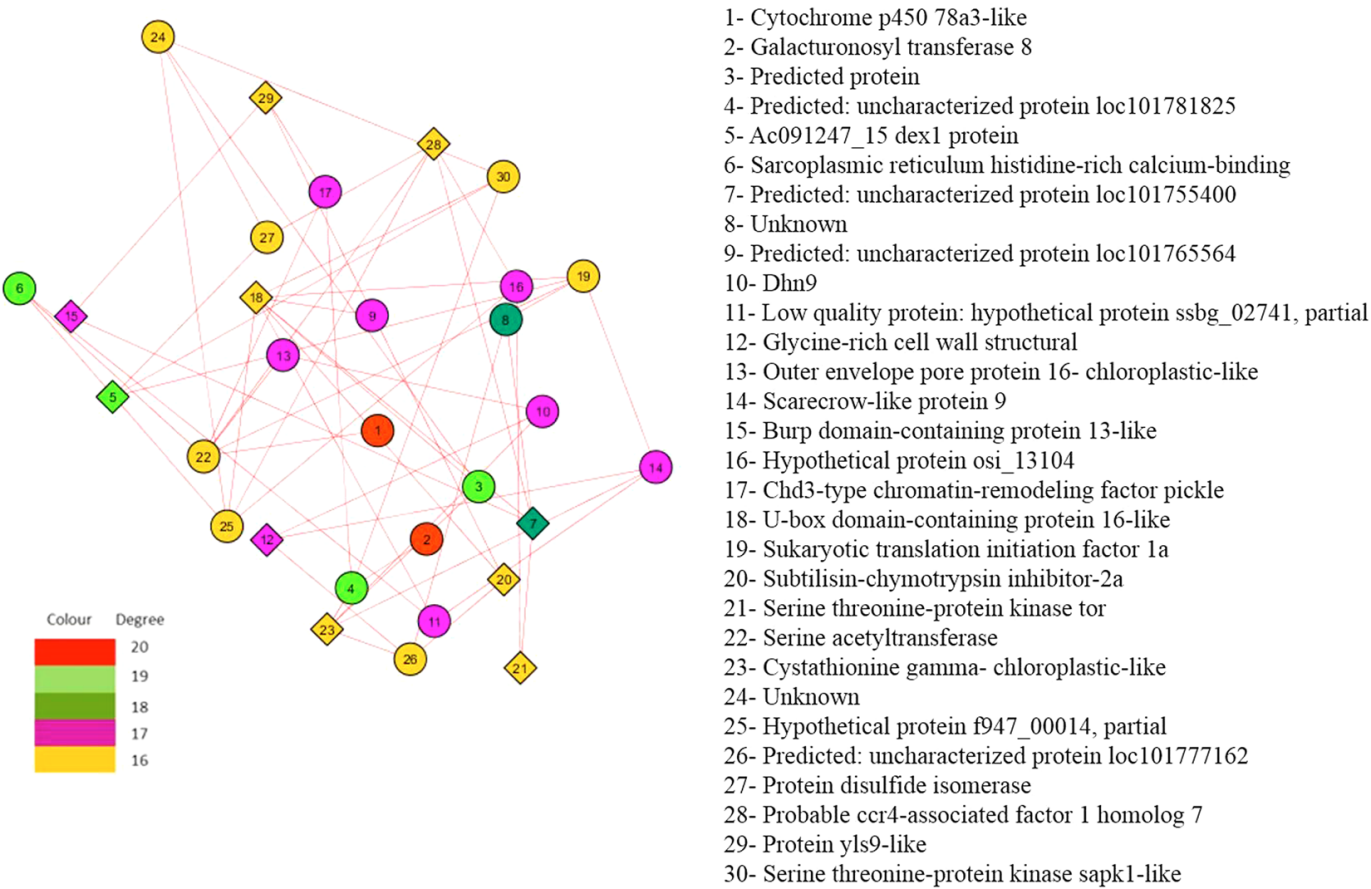
Root Gene Network Analysis: Subnetwork of important Hub gene DEGs.

In root GRN analysis, our dataset reveals response of drought by major intracellular signal transduction mediated by Serine Threonine Protein (STP) kinase which are actually Mitogen Activated Protein Kinases (MAPK)^84^. Drought triggers low expression of this gene to lower MAP Kinase activity. High affinity potential transporter gene was found with lower expression in drought. This genes acts as a sensor for drought in root and also controls uptake of potassium. This gene has been reported to be downregulated in root to balance potassium translocation^60^. Drought leads to high meristematic activity in root mediated by lower expression of O methyltransferase zrp4^85^. Similar low expression of this gene was found in root in response to drought. In drought, ROS gets accumulated in root tip and NADH controls its meristematic activity through ABA pathway by energy balance^86^. This gene was also found differentially regulated in root in response to drought. The details of root GRN hub genes is given in Table 4. Also the leaf subnetwork of important hub DEGs are shown in Fig. 4.

**Table 4:** Description of root hub genes of millet.

Hub Gene	Description/Function	Reference	Status
XP_004974121 cytochrome p450 78a3-like (6 plus)	It mediates senescence associated processes in drought	^99^	Up
XP_009379995 galacturonosyltransferase 8 (9 plus)	It plays role in root tip growth in response to drought	^100^	Up
XP_004981208 ac091247_15 dex1 protein (low)	DEX1 is associated with calcium signalling pathway and low expression (DR) of this in rots inhibits signalling	^101^	Down
XP_004967558 outer envelope pore protein 16- chloroplastic-like(9 Plus)	ABA induced expression of OEP16 protein regulates shuttling amino acids	^102^	Up
XP_002442368 scarecrow-like protein 9 (9 plus)	Scarecrow (SCR) TF is one of the major regulators of plant cellular network in stress mediated by Gibberellic acid	^103^	Up
XP_002437817 eukaryotic translation initiation factor 1a (Negative)	Involved in maintenance of homeostasis in water stress	^104^	Up
EEQ. 24508 serine acetyltransferase (UR/10 Plus)	Involved in recovery of metabolic activity after oxidative inhibition in root tissue	^105^	Up
XP_004970236 protein disulfide isomerase (PDI) (UR /7 plus)	It has been reported to be associated in leaf biomass and leaf size under dehydration stress	^106^	Up
XP_004956826 probable ccr4-associated factor 1 homolog 7 (DR/9 minus)	It plays major role in deadenylation reaction involved in abiotic stress associated with microtubules	^107^	Down
XP_004984154 serine threonine-protein kinase sapk1-like (UR/9plus)	This MAPK family gene is involved in osmosensory signal transduction pathways in osmotic stress	^108^	Up
Serine-Threonine Kinase SAPK1 (Also Known as JNK)

In leaf GRN analysis, our dataset reveals Dehydrin gene family is well known as candidate genes associated with drought tolerance in crop. Among these gene family, in our dataset, we found differential expression of genes *viz*., dehydrin DHN1 and dehydrin COR410-like along with isoform of dehydrin COR410. Dehydrin is known to play role in drought response for crucial protective functions of root tissue. They are produced in response to ABA pathway mediated abiotic stress^87^. This gene family has consensus amino acid seq KIKEKLPG which is reported to be associated with differential expression along with its various isoform in response to drought^88^.

**Figure 5:**
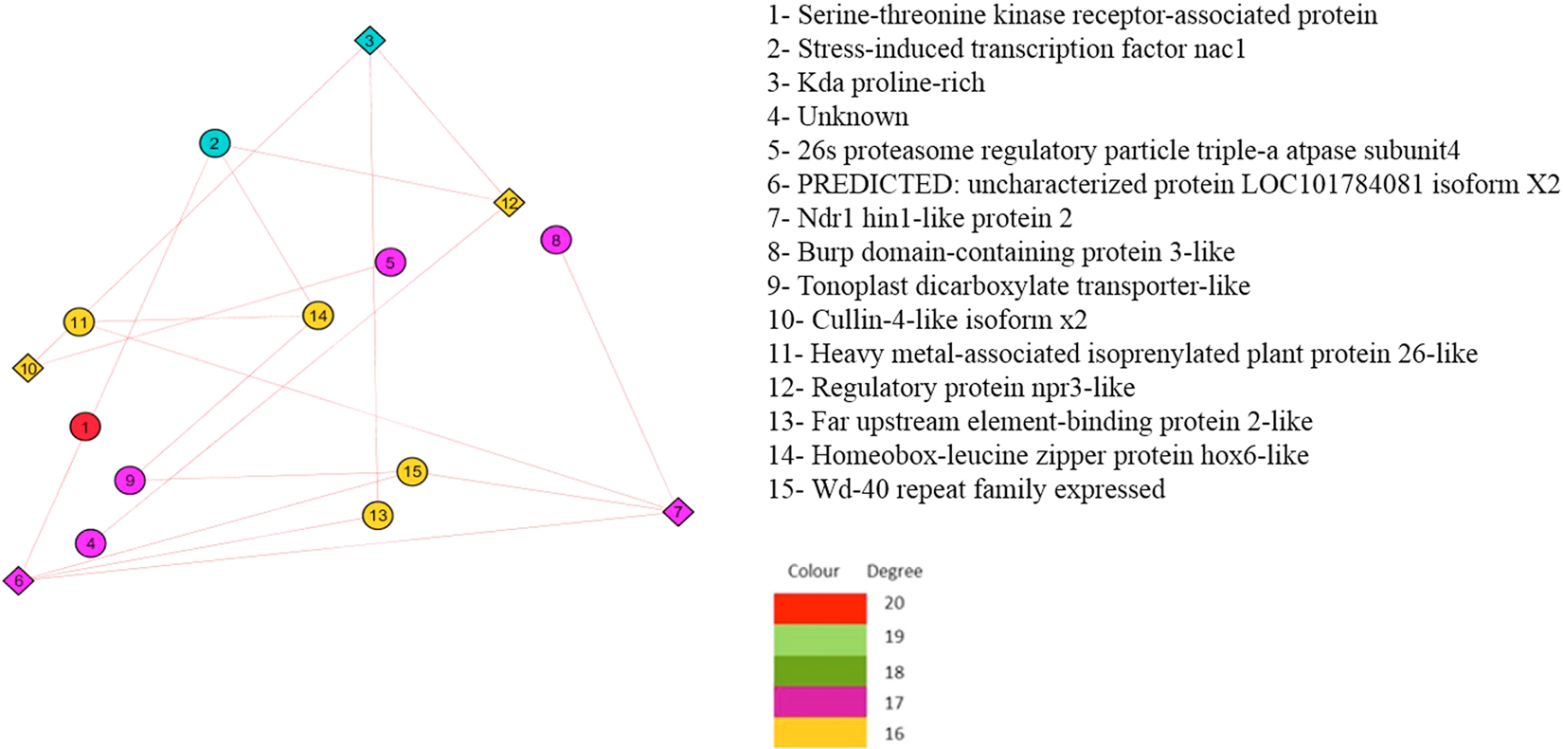
Leaf Gene Network Analysis: Subnetwork of important Hub gene DEGs.

The gene, late embryogenesis abundant protein (LEA) is also reported to be a candidate gene of drought having larger gene family. In our dataset, we found up-regulation of late embryogenesis abundant protein, group 3-like isoform X2, late embryogenesis abundant protein D-34, late embryogenesis abundant protein 3, late embryogenesis abundant protein Lea5. This gene family is reported to play protective role in cells under drought^89^. Two larger gene families, *viz*., dehydrin and LEA were found differentially expressed in our dataset with its isoform. Similar observation has been reported in durum wheat in desiccation stress^90^. The details of leaf GRN hub genes is given in Table 5. Also the leaf subnetwork of important hub DEGs are shown in Fig. 5.

**Table 5:** Description of leaf hub genes of millet.

AFW87112 serine-threonine kinase receptor-associated protein	It mediates ABA dependent pathway of abiotic stress response which is major intracellular signal transduction	^89^	Up
AGU13503 stress-induced transcription factor nac1	Stress-induced transcription factor nac1 plays role in abiotic stress (drought and salt) by modulating ABA mediated pathway	^90^	Up
XP_004977017 (kda proline-rich)	It mediates accumulation of proline in response to drought and salinity	^91^	Down
NP_001148485 26 s proteasome regulatory particle triple-a atpase subunit4	It mediates pathways having antioxidant, photosynthetic and oxidative phosphorylation activities	^92^	Up
XP_004960921 ndr1 hin1-like protein 2	It modulates ABA biosynthesis and signaling pathways in abiotic stress	^93^	Down
XP_004973848 tonoplast dicarboxylate transporter-like	It mediates activity of proton pumps for translocating H + into the vacuoles, thus responsible for accumulation of ions and solutes in response to drought	^95^	Up
XP_004981510 cullin-4-like isoform x2	It represses biochemical activity associated with photomorphogenesis and flowering time under drought stress	^96^	Down
XP_004973957 wd-40 repeat family expressed	This gene family has number of protein repeats which mediates plant secondary metabolism in response to abiotic stimulus	^97^	Up
XP_004951923 achilleol b synthase-like	Achilleol is a type of terpene (secondary metabolite) produced by crop in response to drought in foliar tissue to protect leaves	^98^	Up

### Identification of genic-SSR in Pearl Millet transcriptome

A total of 95017 transcripts were used for SSR mining and 4192 SSRs were obtained. The motif type and their relative abundance are shown in Table 6. Maximum abundance (%) was of trinucleotides repeats (51.62) followed by mononucleotides (24.3), dinucleotides (19.82), tetranucleotides (3.48), pentanucleotides (0.62) and hexanucleotides (0.14). SSRs were also mined from 4 sets of root and leaf tissues and found 385, 244, 618 and 508 markers in (RC, RT), (LC, LT), (RC, LC) and (RT, LT) respectively (Table 6). These putative SSRs markers can be validated by genotyping them in various cultivars. These putative SSRs markers can be validated by genotyping them in various cultivars. Out of 4192 genic region SSRs, we could successfully design primers for 2828 for *de novo* full assembly (Additional file 5). Our reported SSR can be further used as functional domain marker in millet variety improvement program especially with respect to drought tolerance. Such genic region based SSR markers from leaf associated with drought has been reported in mulberry plant^91^. Such genic region SSR markers have an advantage over genomic region, *viz*., transferability, a priory information about gene itself with known functionality. All these are desirable in crop improvement program^92^.

**Table 6:** Detailed s tatistics of identified SSRs.

	*De novo* assembly	(RC, RT)	(LC, LT)	(RC, LC)	(RT, LT)
Total no. of sequences examined	95017	4793	2408	7420	5344
Total no. of identified SSRs	4192	385	244	618	508
No. of SSR containing sequences	3891	345	212	547	452
No. of sequences containing more than 1 SSR	270	32	26	62	47
No. of SSRs present in compound formation	126	16	13	23	20
Mono	1019	87	57	132	116
Di	831	81	49	138	122
Tri	2164	200	129	320	249
Tetra	146	16	8	21	20
Penta	26	1	1	5	1
Hexa	6	0	0	2	0

### SNP and InDel identification

**Figure 6:**
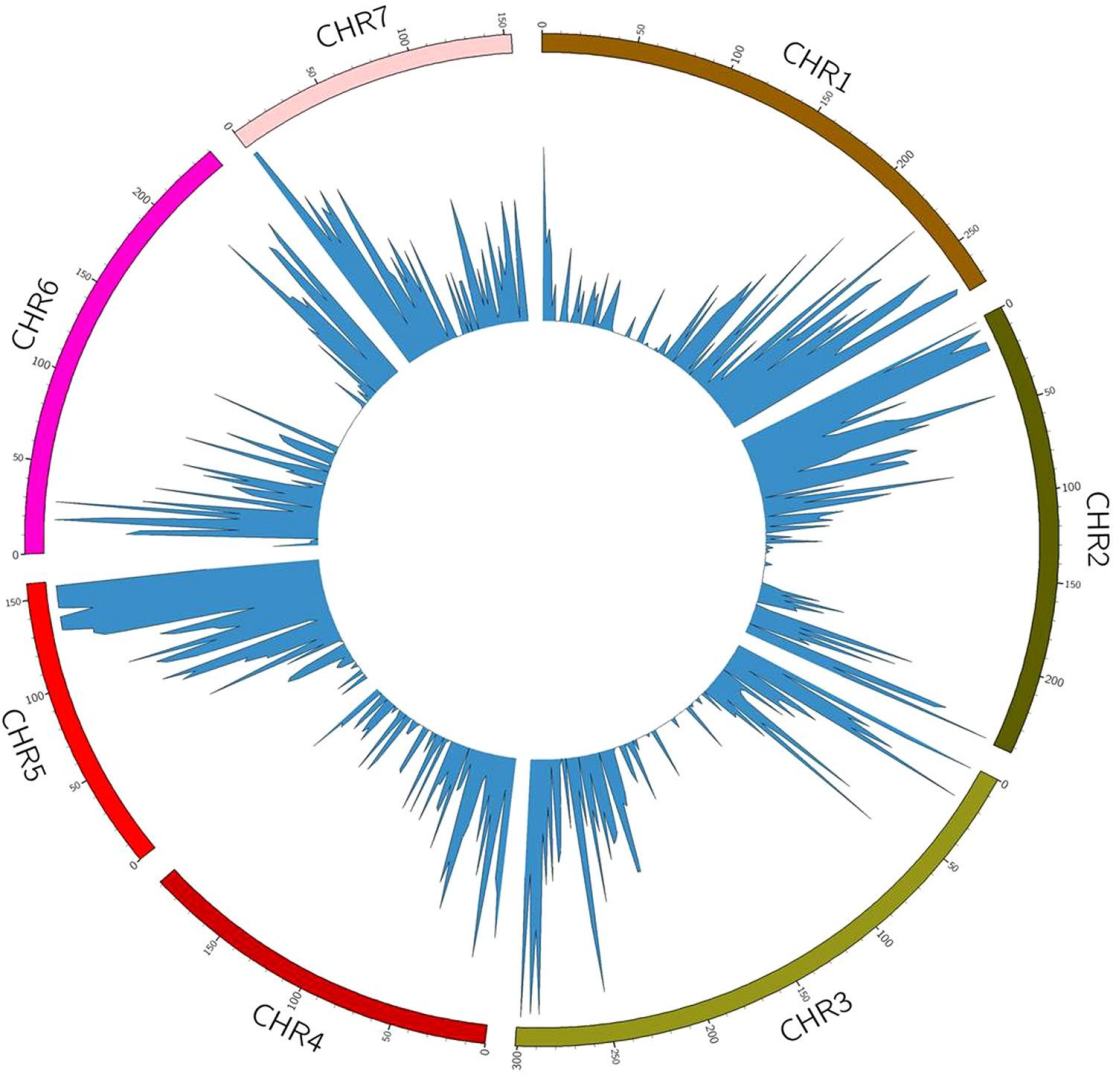
Circular map to depict chromosome-wise SNP distribution among millet genotypes.

SNPs and INDels were discovered successfully by both approaches. Transcriptome based approach using a stringent pipeline, we identified 9318 total variants, having 5587 single nucleotide polymorphisms (SNPs) and 3736 InDels using the SAMtools software (Additional Files 6). In reference based SNP discovery, a total of 18360 SNPs and InDels were obtained. Chromosome wise SNP distribution shows highest SNP over chromosome 2 and lowest number of SNP over chromosome 4 (Table 7). Circular map was also generated to depict chromosome-wise SNP distribution among two genotypes of millet (Fig. 6). More SNPs were found by reference based method than transcriptome based. This is obviously expected as transcriptome based SNP represents intra-genotype variation due to presence of heterozygotes only whereas inter-genotype variation based SNP discovery includes SNPs obtained by alignment of sequences between two genotypes from throughout the genome. This has led to discovery of higher number of SNPs by reference based method. Such alternative alignment has been reported as an efficient method of SNP discovery using two genotypes^93^.

**Table 7:** Chromosome wise distributions of Variants in Pearl millet reference genome.

Chromosome #	Root
1	2697
2	3114
3	2846
4	1652
5	2692
6	2312
7	1959
Unplaced accessions	1088

Putative SNPs in pearl millet transcriptome could be due to millet being predominantly protogynous crop, having high cross pollination resulting into very high heterozygosity also^94^. Being field crop, pearl millet shows high genetic variability within a single open pollinated variety contributing SNP discovery in transcriptome based approach. If the crop has large effective population size and its wind-pollinated reproductive trategy, then there is further increase in heterozygosity yielding these putative SNPs^95^. Besides this, the potential source of SNP could be variation between each of the two sets of 10 plantlets pooled for RNA extraction for transcriptomic data generation. SNPs detected in silico may be either true SNP showing allelic polymorphisms or it may be gene duplication or paralogous or homologous sequence variations^96^. Further observed variants (SNP and InDels) might be due to various other factors such as alignment ambiguity and undetected paralogs^97^. Transcriptome based putative SNPs can be further validated by Sanger sequencing followed by detection of double peak in chromatogram using SNP discovery by heterozygote approach^98^.

These discovered informative SNPs will be of immense use in designing of various throughput genotyping assay using their respective chromosomal location and genomic co-ordinates over reference sequence. Genic region SNP discovery has been used in various crop trait improvement programs like cold tolerance^99^ and dormancy^100^ in wheat, RIL for mapping in *Brassica*^101^, rust resistance in switchgrass^102^, cold tolerance in sorghum^103^ and blister rust in white pine^104^. Such genic region SSRs and SNPs from candidate genes and hub genes of GRN are valuable genomic resource for eQTL discovery, high-density mapping and trait improvement in various crops like common bean^105^ and chickpea^106^.

### Validation and Expression Analysis by qRT-PCR

Relative gene expression value obtained by qRT-PCR analysis of all the 13 genes (6 leaf, 7 root) were in correspondence with computed log fold change value of DEGs (Additional File 7, Fig. 7).

**Figure 7:**
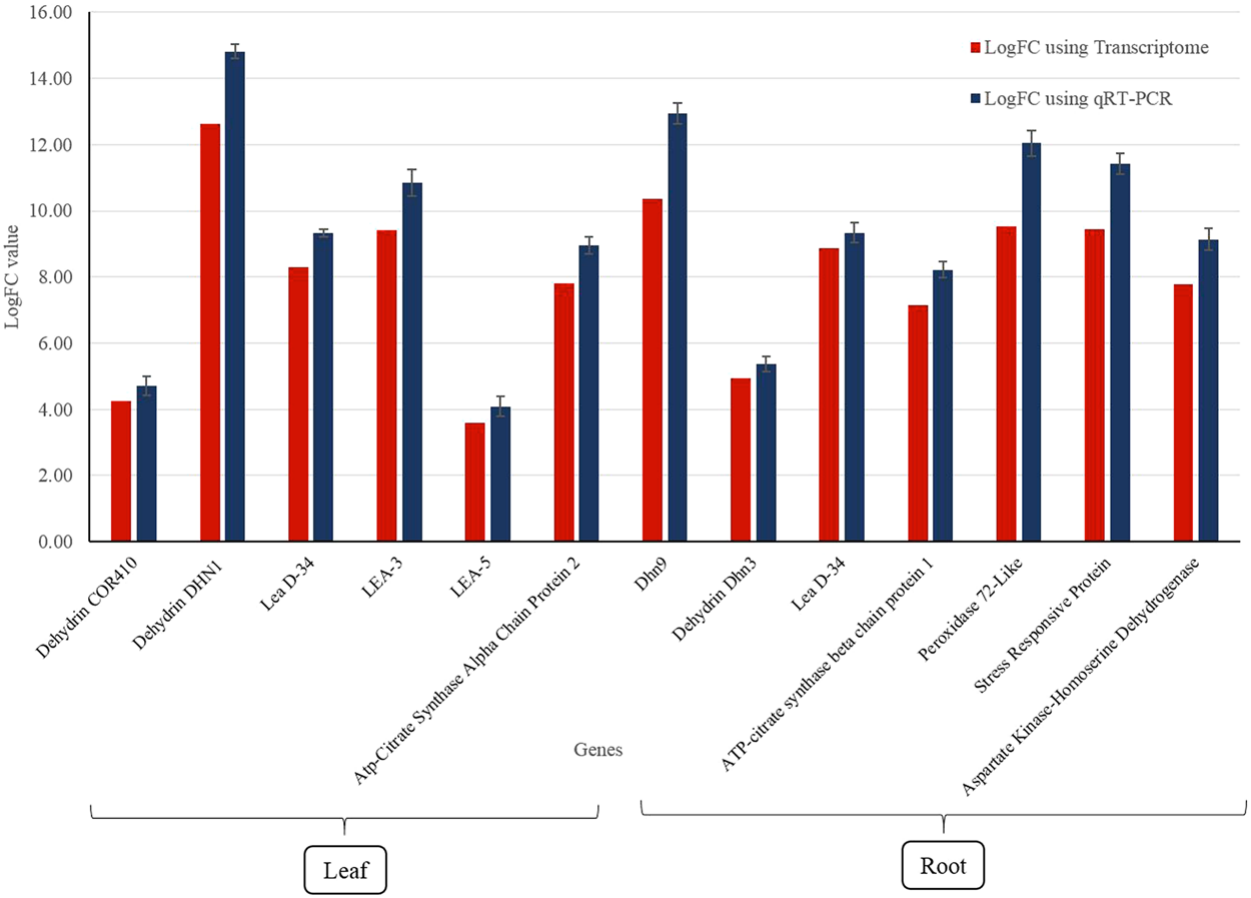
qRT-PCR analysis of randomly selected transcripts.

### Web-genomic resource development

**Figure 8:**
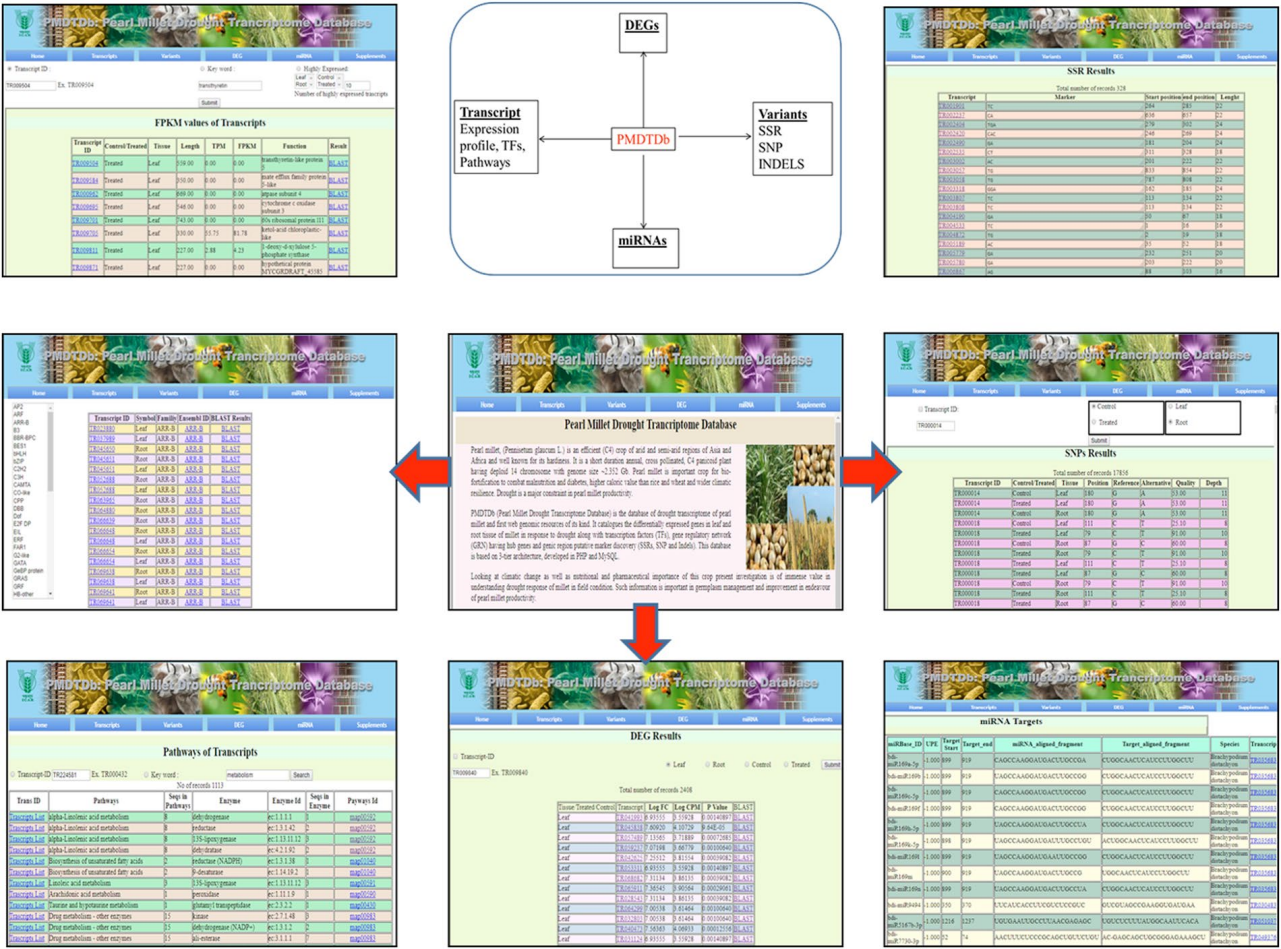
Web interface of *PMDTDb* showing search option for variants, transcripts expression profile and pathways, DEGs and miRNA targets.

Drought associated web genomic resource of pearl millet, *PMDTDb* (http://webtom.cabgrid.res.in/pmdtdb/) has been developed using transcriptome data. It catalogues assembled contigs or transcripts, 19983 DEGs, 7596 transcription factors and a total of 34652 genic region putative markers (SSR markers, SNPs and InDels). The flowchart for its usage is shown in Fig. 8. For other species like foxtail millet, similar genomic resources with molecular markers^107^ and transcription factors database^108^ has been developed. For another species finger millet (*Eleusine coracana* (L.) Gaertn.), such transcriptome sequence has been reported to provides insights into drought tolerance and nutraceutical properties^109^. Based on the present study, we report the first web-based genomic resources of pearl millet. These resources can be used for further in candidate genes-based SNP discovery programs and trait-based association studies in drought improvement.

### Potential application of millet genomic resources

For varietal improvement, traditional breeding program can be supplemented with molecular breeding approach by using genomic resources^110^. Our web-genomic resource is having 17856 SNPs (mined from two millet genotypes) which can be used in variety improvement. Such SNP discovery using even single genotype has been reported in forage crop like *Artemisia*^95^. Since there is no report of SNP discovery in candidate genes associated with drought in millet thus further research can be done by targeted sequencing of the reported genomic resources in large number of varieties and populations. Such approach can reduce the time and cost required for resequencing of large genotypes without prioritizing the candidate genes involved. Similar use of DEG-based SNP discovery has been reported in other crops like wheat for abiotic stress tolerance^94,95^, Switchgrass for biotic stress tolerance^102^. Candidate genes depicted in GRN can be a preferred source of SNP discovery^111^ required for future association studies. Further, such transcriptome-based approach has been reported for eQTL discovery in development of high-density map like *Brassica rapa*^101^.

Our web-based genomic resources is also having 4192 SSRs along with ready to use primers for genotyping can be used in variety identification and improvement program. Similar transcriptome-based SSR has been used in various crops like barley^112^, *S*. *tuberosum*^113^, sugarcane^114^, capsicum^115^, eggplant^116^ and differentiation of Basmati rice from non-Basmati^117^ and also for DUS (Distinctness, Uniformity and Stability) testing for varietal identification^118,119^. Our enlisted SSR can be further used in MAS programme of millet improvement as reported in sorghum^120^, tagging stem rust resistance gene Sr35 in wheat^121^, Saltol QTLs in rice^122^.

Genomic resources of miRNA and its target in *PMDTDb* can be further used in research. It can be useful for both knowledge discovery (mechanism/regulation of drought responsive genes) and application oriented research especially for variety improvement. Since in finger millet, it has been reported that drought tolerance can be increased by use of gene silencing of drought associated miRNA thus our enlisted predicted miRNA can be used for similar work^123^. It has been widely reported that miRNA can be used to enhance crop yield along with increased tolerance to biotic and abiotic stress. Further, genome editing tool like CRISPR-cas9 can be used to control expression of miRNA^124^, thus there is greater scope for further research on enlisted miRNAs/genes by genetic modification for crop improvement. Such genome editing approach has been reported to be very promising in reducing anti-nutrients in millet, thus making it further enriched cereal^125^.

Our enlisted TF genes can be used for future SNP discovery for traits improvement of crop. Similar approach has been reported in oil plant for regulation of dwarfism by negative regulation of DELLA protein^126^. In case of Prunus, traits like the flowering, fruit quality, and biotic and abiotic stress resistance have been found regulated with TF^127^.

## Conclusion

Present work reports root and leaf transcriptomic signature of drought induced by irrigation withdrawal in pearl millet. We found 19983 DEGs, 7596 TFs and GRN having 45 genes controlling drought response. A total of 34652 genic region putative markers *viz*., 4192 SSRs, 12111 SNPs and 6249 InDels are reported. Validation of gene expression by 13 randomly selected genes was in correspondence with computed FPKM values. We report major candidate genes such as LEA, Dhn, ATP-citrate synthase family, peroxidase, stress responsive protein and Aspartate Kinase-Homoserine Dehydrogenase genes. Enlisted candidate genes can be used for further SNP discovery programs and association studies. Looking at climatic change and nutritional and pharmaceutical value of this crop as well as its genetic potential of resilience, present investigation is of immense value in understanding drought response of millet in field condition. Such information is important in germplasm management and improvement in endeavour of pearl millet productivity.

## Electronic supplementary material

Supplementary Figures 1 & 2

Additional file 1

Additional file 2

Additional file 3

Additional file 4

Additional file 5

Additional file 6

Additional file 7

## References

[1] Varshney RK (2017). Pearl millet genome sequence provides a resource to improve agronomic traits in arid environments. Nature Biotechnology..

[2] Patil KB, Chimmad BV, Itagi S (2015). Glycemic index and quality evaluation of little millet (Panicum miliare) flakes with enhanced shelf life. Journal of food science and technology..

[3] FAOSTAT: Production, Crops, Millet, data”. Food and Agriculture Organization. 2011. Archived from the original on 2013-01-14. (2010).

[4] Saleh AS, Zhang Q, Chen J, Shen Q (2013). Millet grains: nutritional quality, processing, and potential health benefits. Comprehensive Reviews in Food Science and Food Safety..

[5] Nambiar VS, Dhaduk JJ, Sareen N, Shahu T, Desai R (2011). Potential functional implications of pearl millet (Pennisetum glaucum) in health and disease. Journal of Applied Pharmaceutical Science..

[6] ICRISAT, P. Alternative Uses of Sorghum and Pearl Millet in Asia. (2003).

[7] Sloan AE (1999). Positive eating and problem treating: Nutraceuticals and cereal-based foods in the 21st century. Cereal foods world..

[8] Dai A (2013). Increasing drought under global warming in observations and models. Nature Climate Change..

[9] Choudhary M, Jayanand, Padaria JC (2015). Transcriptional profiling in pearl millet (*Pennisetum glaucum L*.*R*. *Br*.) for identification of differentially expressed drought responsive genes. Physiology and Molecular Biology of Plants..

[10] Habiyaremye, C. *et al*. Proso Millet (Panicum miliaceum L.) and Its Potential for Cultivation in the Pacific Northwest, US: A Review. *Frontiers in plant science*. **7** (2016). 10.3389/fpls.2016.01961 PMC5220228 28119699

[11] Yadav RS, Sehgal D, Vadez V (2010). Using genetic mapping and genomics approaches in understanding and improving drought tolerance in pearl millet. Journal of Experimental Botany..

[12] Qi X (2001). Development of simple sequence repeat markers from bacterial artificial chromosomes without subcloning. Biotechniques..

[13] James D (2015). Development and characterization of a high temperature stress responsive subtractive cDNA library in Pearl Millet Pennisetum glaucum (L.) R. Br. J Exp Biol..

[14] Rajaram V (2013). Pearl millet [Pennisetum glaucum (L.) R. Br.] consensus linkage map constructed using four RIL mapping populations and newly developed EST-SSRs. BMC genomics..

[15] Senthilvel S (2008). Development and mapping of simple sequence repeat markers for pearl millet from data mining of expressed sequence tags. BMC Plant Biology..

[16] Yadav OP, Mitchell SE, Zamora A, Fulton TM, Kresovich S (2007). Development of new simple sequence repeat markers for pearl millet. Journal of SAT Agricultural Research..

[17] Budak H, Pedraza F, Cregan PB, Baenziger PS, Dweikat I (2003). Development and utilization of SSRs to estimate the degree of genetic relationships in a collection of pearl millet germplasm. Crop Science..

[18] Jogaiah S, Anand Kumar S, Thakur RP, Rao VP, Shekar SH (2009). Molecular characterization of Sclerospora graminicola, the incitant of pearl millet downy mildew revealed by ISSR markers. J. Phytopath..

[19] Jogaiah S, Sharathchandra RG, Niranjan Raj., Vedamurthy AB, Shekar SH (2014). Development of SCAR marker associated with downy mildew disease resistance in pearl millet (Pennisetum glaucum L.). Molecular Biology Reports..

[20] Sehgal D (2012). Integration of gene-based markers in a pearl millet genetic map for identification of candidate genes underlying drought tolerance quantitative trait loci. BMC Plant Biology..

[21] Supriya A (2011). Development of a molecular linkage map of pearl millet integrating DArT and SSR markers. Theoretical and Applied Genetics..

[22] Serba, D. D., & Yadav, R. S. Genomic tools in pearl millet breeding for drought tolerance: status and prospects. *Frontiers in plant science*. **7** (2016). 10.3389/fpls.2016.01724 PMC5118443 27920783

[23] Shivhare, R., & Lata, C. Exploration of genetic and genomic resources for abiotic and biotic stress tolerance in pearl millet. *Frontiers in plant science*. **7** (2016). 10.3389/fpls.2016.02069 PMC5253385 28167949

[24] Huang, C. Y. *et al*. A DNA-based method for studying root responses to drought in field-grown wheat genotypes. *Scientific reports*. **3** (2013). 10.1038/srep03194 PMC3824167 24217242

[25] Janiak A, Kwaśniewski M, Szarejko I (2016). Gene expression regulation in roots under drought. Journal of experimental botany..

[26] Brunner, I., Herzog, C., Dawes, M. A., Arend, M. & Sperisen, C. How tree roots respond to drought. *Frontiers in Plant Science*. **6** (2015). 10.3389/fpls.2015.00547 PMC4518277 26284083

[27] Larson JE, Funk JL (2016). Seedling root responses to soil moisture and the identification of a belowground trait spectrum across three growth forms. New Phytologist..

[28] Djanaguiraman, M., Perumal, R., Ciampitti, I. A., Gupta, S. K. & Prasad, P. V. V. Quantifying pearl millet response to high temperature stress: thresholds, sensitive stages, genetic variability and relative sensitivity of pollen and pistil. *Plant*, *cell & environment*., 10.1111/pce.12931 (2017). 10.1111/pce.12931 28173611

[29] Liu SC (2016). Transcriptomic analysis of tea plant responding to drought stress and recovery. PloS one..

[30] Iovieno, P. *et al*. Transcriptomic changes drive physiological responses to progressive drought stress and rehydration in tomato. *Frontiers in plant science*. **7** (2016). 10.3389/fpls.2016.00371 PMC4814702 27066027

[31] Fu L (2016). Physiological investigation and transcriptome analysis of polyethylene glycol (PEG)-induced dehydration stress in cassava. International journal of molecular sciences..

[32] Shin JH (2015). Transcriptomic changes due to water deficit define a general soybean response and accession-specific pathways for drought avoidance. BMC plant biology.

[33] Jain D, Chattopadhyay D (2010). Analysis of gene expression in response to water deficit of chickpea (Cicer arietinum L.) varieties differing in drought tolerance. BMC plant biology..

[34] Tadele, Z. Drought adaptation in millets. In *Abiotic and Biotic Stress in Plants-Recent Advances and Future Perspectives*. InTech., 10.5772/61929 (2016).

[35] Zou C, Wang P, Xu Y (2016). Bulked sample analysis in genetics, genomics and crop improvement. Plant biotechnology journal..

[36] Accerbi, M. *et al*. Plant MicroRNAs, Methods in Molecular Biology (eds Meyers, B. C. & Green, P. J.) **592 (**Humana Press, 2010).

[37] Bolger AM, Lohse M, Usadel B (2014). Trimmomatic: a flexible trimmer for Illumina sequence data. Bioinformatics.

[38] Haas, B. J. *et al*. De novo transcript sequence reconstruction from RNA-Seq: reference generation and analysis with Trinity. *Nature protocols*. **8** (2013). 10.1038/nprot.2013.084 PMC3875132 23845962

[39] Li B, Dewey CN (2011). RSEM: accurate transcript quantification from RNA-Seq data with or without a reference genome. BMC bioinformatics..

[40] Robinson MD, McCarthy DJ, Smyth G (2010). K. edgeR: a Bioconductor package for differential expression analysis of digital gene expression data. Bioinformatics..

[41] Yendrek CR, Ainsworth EA, Thimmapuram J (2012). The bench scientist’s guide to statistical analysis of RNA-Seq data. BMC research notes..

[42] Conesa, A. & Götz, S. Blast2GO: A comprehensive suite for functional analysis in plant genomics. *International journal of plant genomics*. **2008** (2008). 10.1155/2008/619832 PMC2375974 18483572

[43] Pérez-Rodríguez P (2009). PlnTFDB: updated content and new features of the plant transcription factor database. Nucleic acids research..

[44] Dai X, Zhao P (2011). X. psRNATarget: a plant small RNA target analysis server. Nucleic acids research..

[45] Shannon P (2003). Cytoscape: a software environment for integrated models of biomolecular interaction networks. Genome research..

[46] Thiel, T. MISA—Microsatellite identification tool. http://pgrc.ipk-gatersleben.de/misa/ (2003).

[47] Untergasser A (2012). Primer3—new capabilities and interfaces. Nucleic acids research..

[48] Krzywinski M (2009). Circos: an information aesthetic for comparative genomics. Genome Res..

[49] Li H (2009). The sequence alignment/map format and SAMtools. Bioinformatics..

[50] Kujur A (2015). Employing genome-wide SNP discovery and genotyping strategy to extrapolate the natural allelic diversity and domestication patterns in chickpea. Front Plant Sci..

[51] Helyar SJ (2012). SNP Discovery Using Next Generation Transcriptomic Sequencing in Atlantic Herring (Clupea harengus). Plos One..

[52] Livak KJ, Schmittgen TD (2001). Analysis of relative gene expression data using real-time quantitative PCR and the 2− ΔΔCT method. Methods..

[53] Watanabe S (2014). The purine metabolite allantoin enhances abiotic stress tolerance through synergistic activation of abscisic acid metabolism. Plant, cell & environment..

[54] Rai VK (2002). Role of amino acids in plant responses to stresses. Biologia Plantarum..

[55] Ding Y, Zhou X, Zuo L, Wang H, Yu D (2016). Identification and functional characterization of the sulfate transporter gene GmSULTR1; 2b in soybean. BMC genomics..

[56] Kishor PK (2005). Regulation of proline biosynthesis, degradation, uptake and transport in higher plants: its implications in plant growth and abiotic stress tolerance. Current science..

[57] Blanco FA, Meschini EP, Zanetti ME, Aguilar OM (2009). A small GTPase of the Rab family is required for root hair formation and preinfection stages of the common bean–Rhizobium symbiotic association. The Plant Cell..

[58] Caverzan A (2012). Plant responses to stresses: role of ascorbate peroxidase in the antioxidant protection. Genetics and molecular biology..

[59] Yancey PH, Clark ME, Hand SC, Bowlus RD, Somero GN (1982). Living with water stress: Evolution of osmolyte system. Science..

[60] Wang TB, Gassmann W, Rubio F, Schroeder JI, Glass AD (1998). Rapid up-regulation of HKT1, a high-affinity potassium transporter gene, in roots of barley and wheat following withdrawal of potassium. Plant Physiology..

[61] Durand M (2016). Water deficit enhances C export to the roots in Arabidopsis thaliana plants with contribution of sucrose transporters in both shoot and roots. Plant physiology..

[62] Sofo A, Dichio B, Xiloyannis C, Masia A (2005). Antioxidant defences in olive trees during drought stress: changes in activity of some antioxidant enzymes. Functional Plant Biology..

[63] Kawashima CG, Berkowitz O, Hell R, Noji M, Saito K (2005). Characterization and expression analysis of a serine acetyltransferase gene family involved in a key step of the sulfur assimilation pathway in Arabidopsis. Plant Physiology..

[64] Guo P (2009). Differentially expressed genes between drought-tolerant and drought-sensitive barley genotypes in response to drought stress during the reproductive stage. Journal of experimental botany..

[65] Zhao, P. *et al*. New insights on drought stress response by global investigation of gene expression changes in Sheepgrass (Leymus chinensis). *Frontiers in plant science*. **7** (2016). 10.3389/fpls.2016.00954 PMC4928129 27446180

[66] Umezawa T, Yoshida R, Maruyama K, Yamaguchi-Shinozaki K, Shinozaki K (2004). SRK2C, a SNF1-related protein kinase 2, improves drought tolerance by controlling stress-responsive gene expression in Arabidopsis thaliana. Proceedings of the National Academy of Sciences of the United States of America..

[67] Tang, X., Wang, H., Chu, L., & Shao, H. KvLEA, a New Isolated Late Embryogenesis Abundant Protein Gene from Kosteletzkya virginica Responding to Multiabiotic Stresses. *BioMed research international*. **2016** (2016). 10.1155/2016/9823697 PMC4829701 27123459

[68] Merewitz E, Xu Y, Huang B (2016). Differentially Expressed Genes Associated with Improved Drought Tolerance in Creeping Bentgrass Overexpressing a Gene for Cytokinin Biosynthesis. PloS one..

[69] Cramer GR (2013). Proteomic analysis indicates massive changes in metabolism prior to the inhibition of growth and photosynthesis of grapevine (Vitis vinifera L.) in response to water deficit. BMC Plant Biology..

[70] Chmielewska, K. *et al*. Analysis of drought-induced proteomic and metabolomic changes in barley (Hordeum vulgare L.) leaves and roots unravels some aspects of biochemical mechanisms involved in drought tolerance. *Frontiers in plant science*. **7** (2016). 10.3389/fpls.2016.01108 PMC4962459 27512399

[71] NIU CF (2012). Wheat WRKY genes TaWRKY2 and TaWRKY19 regulate abiotic stress tolerance in transgenic Arabidopsis plants. Plant, cell & environment.

[72] McGrann GR (2015). Contribution of the drought tolerance‐related Stress‐responsive NAC1 transcription factor to resistance of barley to Ramularia leaf spot. Molecular plant pathology..

[73] Wagner A (1999). Genes regulated cooperatively by one or more transcription factors and their identification in whole eukaryotic genomes. Bioinformatics.

[74] Ji Z (2012). The Forkhead Transcription Factor FOXK2 Promotes AP-1-Mediated Transcriptional Regulation. Mol. Cell. Biol..

[75] Boeva V (2016). Analysis of Genomic Sequence Motifs for Deciphering Transcription Factor Binding and Transcriptional Regulation in Eukaryotic Cells. Front. Genet..

[76] Cui Q, Yu Z, Pan Y, Purisima EO, Wang E (2007). MicroRNAs preferentially target the genes with high transcriptional regulation complexity. Biochem. Biophys. Res. Commun..

[77] Zeng C (2010). Conservation and divergence of microRNAs and their functions in Euphorbiaceous plants. Nucleic Acids Res..

[78] Hon LS, Zhang Z (2007). The roles of binding site arrangement and combinatorial targeting in microRNA repression of gene expression. Genome Biol..

[79] Bakhshi B (2017). The contrasting microRNA content of a drought tolerant and a drought susceptible wheat cultivar. J. Plant Physiol..

[80] Zhang JW, Long Y, Xiao XG, Pei XW (2017). Identification of microRNAs in Response to Drought in Common Wild Rice (Oryza rufipogon Griff). Shoots and Roots. PloS one..

[81] Xie F, Wang Q, Sun R, Zhang B (2015). Deep sequencing reveals important roles of microRNAs in response to drought and salinity stress in cotton. J. Exp. Bot..

[82] Katiyar A (2015). Identification of novel drought-responsive microRNAs and trans-acting siRNAs from Sorghum bicolor (L.) Moench by high-throughput sequencing analysis. Front. Plant Sci..

[83] De Lima JC, Loss-Morais G, Margis R (2012). MicroRNAs play critical roles during plant development and in response to abiotic stresses. Genet. Mol. Biol..

[84] Hirt H (1997). Multiple roles of MAP kinases in plant signal transduction. Trends in Plant Science..

[85] Held BM, Wang H, John I, Wurtele ES, Colbert JT (1993). An mRNA putatively coding for an O-methyltransferase accumulates preferentially in maize roots and is located predominantly in the region of the endodermis. Plant Physiology..

[86] Yang L (2014). ABA-mediated ROS in mitochondria regulate root meristem activity by controlling PLETHORA expression in Arabidopsis. PLoS genetics..

[87] Hassan NM, El-Bastawisy ZM, El-Sayed AK, Ebeed HT, Alla MMN (2015). Roles of dehydrin genes in wheat tolerance to drought stress. Journal of advanced research..

[88] Close TJ, Fenton RD, Moonan F (1993). A view of plant dehydrins using antibodies specific to the carboxy terminal peptide. Plant molecular biology..

[89] Gao J, Lan T (2016). Functional characterization of the late embryogenesis abundant (LEA) protein gene family from Pinus tabuliformis (Pinaceae) in Escherichia coli. Scientific reports..

[90] Nezhadahmadi, A., Prodhan, Z. H., & Faruq, G. Drought tolerance in wheat. *The Scientific World Journal*. **2013** (2013). 10.1155/2013/610721 PMC3844267 24319376

[91] Thumilan BM (2016). Development and Characterization of Genic SSR Markers from Indian Mulberry Transcriptome and Their Transferability to Related Species of Moraceae. PloS one.

[92] Shingane, S. N. Comparative Advantages of Genetic-SSRs Over Genomic SSRs for Crop Improvement. http://www.biotecharticles.com/Genetics-Article/Comparative-Advantages-of-Genic-SSRs-Over-Genomic-SSRs-for-Crop-Improvement-3258.html (2014).

[93] Barbazuk WB, Schnable PS (2011). SNP discovery by transcriptome pyrosequencing. Methods Mol Biol..

[94] Patil, J. V. *Milets and Sorghum: Biology and Genetic Improvement*. 1–504 (Wiley, 2016).

[95] Bajgain P, Richardson BA, Price JC, Cronn RC, Udall JA (2011). Transcriptome characterization and polymorphism detection between subspecies of big sagebrush (Artemisia tridentata). BMC Genomics.

[96] Chagné D (2008). Development of a set of SNP markers present in expressed genes of the apple. Genomics.

[97] Mansueto L (2016). SNP-Seek II: A resource for allele mining and analysis of big genomic data in Oryza sativa. Curr. Plant Biol..

[98] He B, Li Y, Ni Z, Xu L (2017). Transcriptome sequencing and SNP detection in Phoebe chekiangensis. PeerJ.

[99] Laudencia-Chingcuanco D (2011). Genome-wide gene expression analysis supports a developmental model of low temperature tolerance gene regulation in wheat (Triticum aestivum L.). BMC genomics..

[100] Barrero JM (2015). Transcriptomic analysis of wheat near-isogenic lines identifies PM19-A1 and A2 as candidates for a major dormancy QTL. Genome biology..

[101] Devisetty UK, Covington MF, Tat AV, Lekkala S, Maloof JN (2014). Polymorphism identification and improved genome annotation of Brassica rapa through deep RNA sequencing. G3: Genes, Genomes, Genetics..

[102] Serba, D. D. *et al*. Transcriptome profiling of rust resistance in switchgrass using RNA-Seq analysis. *The Plant Genome*. **8** (2015). 10.3835/plantgenome2014.10.0075 33228298

[103] Chopra R (2015). Transcriptome profiling and validation of gene based single nucleotide polymorphisms (SNPs) in sorghum genotypes with contrasting responses to cold stress. BMC genomics..

[104] Liu JJ, Sniezko RA, Sturrock RN, Chen H (2014). Western white pine SNP discovery and high-throughput genotyping for breeding and conservation applications. BMC plant biology..

[105] Wu J, Wang L, Li L, Wang S (2014). De novo assembly of the common bean transcriptome using short reads for the discovery of drought-responsive genes. PLoS One..

[106] Srivastava R, Bajaj D, Malik A, Singh M, Parida SK (2016). Transcriptome landscape of perennial wild Cicer microphyllum uncovers functionally relevant molecular tags regulating agronomic traits in chickpea. Scientific reports..

[107] VS B, Muthamilarasan M, Misra G, Prasad M (2012). FmMDb: a versatile database of foxtail millet markers for millets and bioenergy grasses research. PloS one..

[108] Bonthala VS, Muthamilarasan M, Roy R, Prasad M (2014). FmTFDb: a foxtail millet transcription factors database for expediting functional genomics in millets. Molecular biology reports..

[109] Hittalmani S (2017). Genome and Transcriptome sequence of Finger millet (*Eleusine coracana* (L.) Gaertn.) provides insights into drought tolerance and nutraceutical properties. BMC Genomics..

[110] Collard BCY, Mackill DJ (2008). Marker-assisted selection: an approach for precision plant breeding in the twenty-first century. Philos. Trans. R. Soc. Lond. B. Biol. Sci..

[111] Kim, D.-C., Wang, J., Liu, C. & Gao, J. Inference of SNP-gene regulatory networks by integrating gene expressions and genetic perturbations. *Biomed Res*. *Int*. 629697 (2014). 10.1155/2014/629697 PMC4127230 25136606

[112] Karakousis A (2003). Potential of SSR markers for plant breeding and variety identification in Australian barley germplasm. Aust. J. Agric. Res.

[113] Kawchuk LM, Martin RF, Mcpherson. J (1990). Resistance in transgenic potato expressing the potato leafroll virus coat protein gene. Mol Plant Microbe.

[114] Manigbas NL, Villegas LC (2004). Microsatellite Markers in Hybridity tests to identify true hybrids of sugarcane. Philipp J Crop Sci..

[115] Shirasawa K (2013). Development of Capsicum EST-SSR markers for species identification and in silico mapping onto the tomato genome sequence. Mol. Breed..

[116] Stàgel A, Portis E, Toppino L, Rotino G, Lanteri S (2008). Gene-based microsatellite development for mapping and phylogeny studies in eggplant. BMC Genomics.

[117] Archak S, Lakshminarayanareddy V, Nagaraju J (2007). High-throughput multiplex microsatellite marker assay for detection and quantification of adulteration in Basmati rice (Oryza sativa). Electrophoresis.

[118] McCouch SR (1997). Microsatellite marker development, mapping and applications in rice genetics and breeding. Plant Mol. Biol..

[119] Becher SA (2000). Microsatellites for cultivar identification in Pelargonium. TAG. Theor. Appl. Genet..

[120] Wang Y-H, Bible P, Loganantharaj R, Upadhyaya HD (2012). Identification of SSR markers associated with height using pool-based genome-wide association mapping in sorghum. Mol. Breed..

[121] Babiker E, Ibrahim AMH, Yen Y, Stein J (2009). Identification of a microsatellite marker associated with stem rust resistance gene Sr35 in wheat. Aust. J. Crop Sci..

[122] Thomson MJ (2010). Characterizing the Saltol Quantitative Trait Locus for Salinity Tolerance in Rice. Rice.

[123] Ramegowda V (2017). GBF3 transcription factor imparts drought tolerance in Arabidopsis thaliana. Sci. Rep..

[124] Djami-Tchatchou AT, Sanan-Mishra N, Ntushelo K, Dubery IA (2017). Functional Roles of microRNAs in Agronomically Important Plants—Potential as Targets for Crop Improvement and Protection. Front. Plant Sci..

[125] Vinoth, A., & Ravindhran, R. Biofortification in Millets: A Sustainable Approach for Nutritional Security. *Front*. *Plant Sci*. **8** (2017). 10.3389/fpls.2017.00029 PMC5253353 28167953

[126] Rahman S, Vasu A, Gangaraj KP, Hemalatha N, Rajesh MK (2015). Structural basis for recognition of Gibberellin by its receptor GID1 (GA-INSENSITIVE DWARF1) in Oil Palm. Int. J. Innov. Res. Comput. Commun. Eng..

[127] Bianchi VJ (2015). Prunus transcription factors: breeding perspectives. Front. Plant Sci..

