# Author Correction: Transcriptomic signature of drought response in pearl millet (*Pennisetum glaucum* (L.) and development of web-genomic resources

**DOI:** 10.1038/s41598-018-25622-2

**Published:** 2018-05-11

**Authors:** Sarika Jaiswal, Tushar J. Antala, M. K. Mandavia, Meenu Chopra, Rahul Singh Jasrotia, Rukam S. Tomar, Jashminkumar Kheni, U. B. Angadi, M. A. Iquebal, B. A. Golakia, Anil Rai, Dinesh Kumar

**Affiliations:** 1 Centre for Agricultural Bioinformatics (CABin), ICAR-Indian Agricultural Statistics Research Institute, New Delhi, India; 2 Department of Biochemistry and Biotechnology, Junagadh Agricultural University, Junagadh, Gujarat India

Correction to: *Scientific Reports* 10.1038/s41598-018-21560-1, published online 21 February 2018

The original version of this Article contained an error in the order of the Figures. Figures 6, 7 and 8 were published as 8, 6 and 7.  

This has now been corrected in the PDF and HTML versions of this Article.

