# Supplementary material for: Transcriptomic signature of drought response in pearl millet (Pennisetum glaucum (L.) and development of web-genomic resources
Source: Sci Rep. 2018 Feb 21;8:3382. doi: 10.1038/s41598-018-21560-1 (PMC5821703; doi:10.1038/s41598-018-21560-1)
Supplement: Supplementary file 1 — Supplementary Figures 1 & 2 [file 41598_2018_21560_MOESM1_ESM.pdf]

Transcriptomic signature of drought response in pearl millet (*Pennisetum glaucum* (L.) and development of web-genomic resources

Sarika Jaiswal, Tushar J. Antala, M. K. Mandavia, Meenu Chopra, Rahul Singh Jasrotia, Rukam S. Tomar, Jashminkumar Kheni, U.B. Angadi, MA Iquebal, B. A. Golakia, Anil Rai, Dinesh Kumar

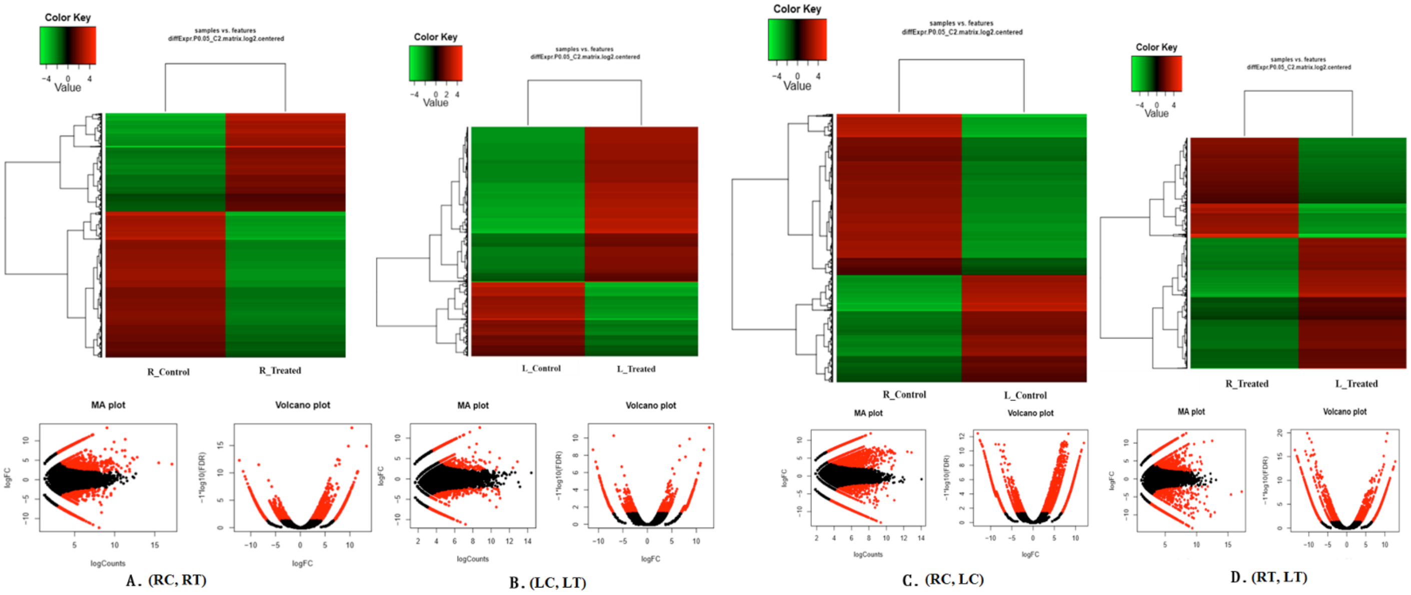

Supplementary Figures 1 (A, B, C, D). Heat maps, MA-plots and Volcano plots showing the relative expression levels of each transcript in four combinations of dataset. Up-regulated genes are shown by red and down-regulated by green color.

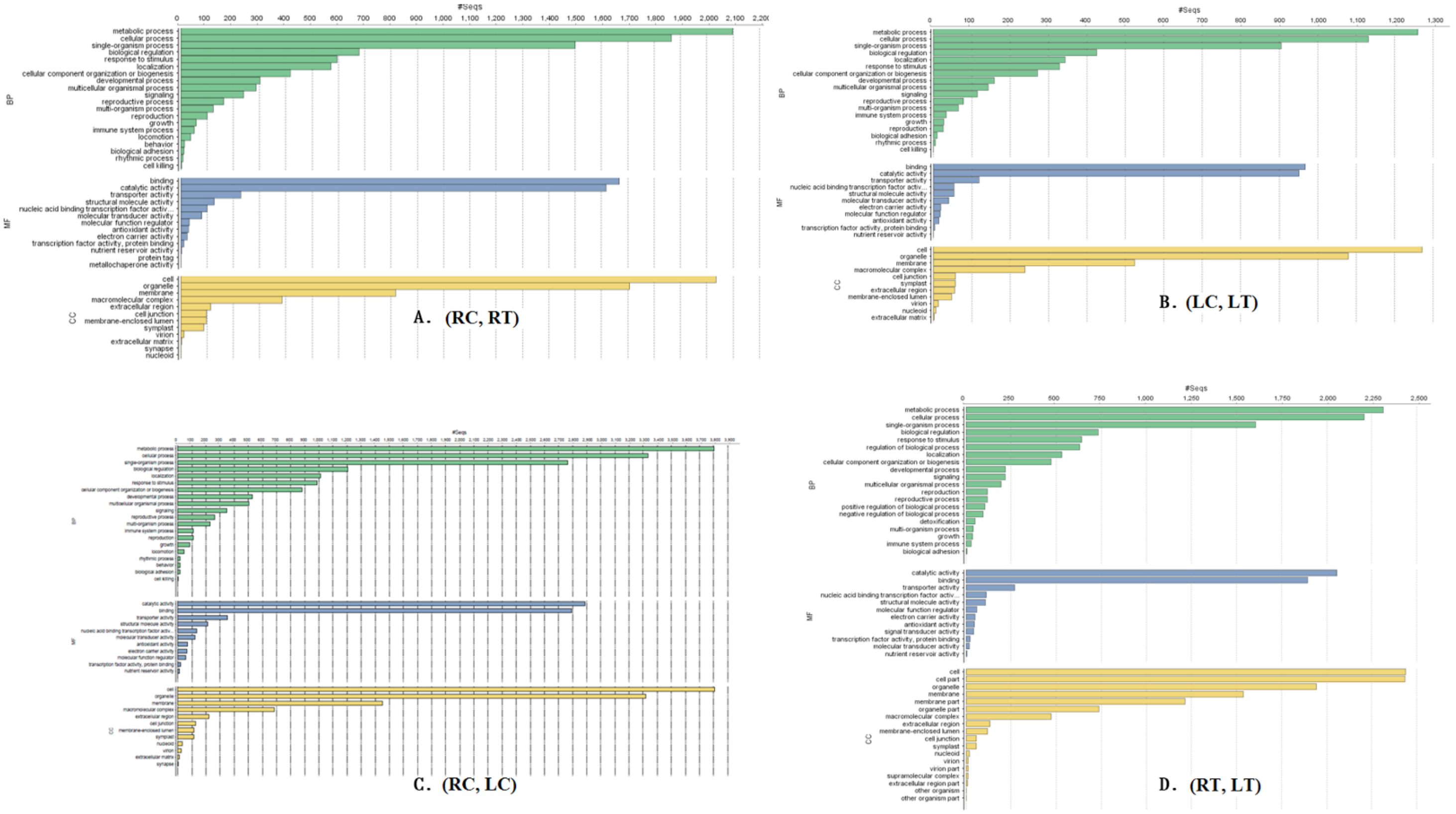

Supplementary Figures 2 (A, B, C and D). Gene ontology classification delineating biological processes, molecular functions and cellular components in in four combinations of dataset.

Additional Files' Legends:

Additional file 1. List of significant differentially expressed genes in millet transcriptome

Additional file 2. Pathways identified in millet root and leaf transcriptome associated with drought

Additional File 3. Blast hits of identified transcription factors in millet transcriptome

Additional File 4. Blast results of identified miRNA with their targets in millet transcriptome

Additional File 5. Identified putative SSRs and its primers in millet transcriptome

Additional File 6. Identified SNPs in millet transcriptome

Additional File 7. qRT-PCR analysis of randomly selected transcripts
